# Testing individual baroreflex responses to hypoxia-induced peripheral chemoreflex stimulation

**DOI:** 10.1007/s10286-019-00660-6

**Published:** 2020-01-23

**Authors:** Hendrik Kronsbein, Darius A. Gerlach, Karsten Heusser, Alex Hoff, Fabian Hoffmann, André Diedrich, Heimo Ehmke, Jens Jordan, Jens Tank

**Affiliations:** 1 Department of Cardiovascular Aerospace Medicine, Institute of Aerospace Medicine, German Aerospace Center (DLR), 51147 Cologne, Germany; 2 Institute of Cellular and Integrative Physiology, University Medical Center Hamburg-Eppendorf, Hamburg, Germany; 3 Division of Cardiology, Angiology and Pneumology, University Hospital Cologne, Cologne, Germany; 4 Division of Medicine, Division of Clinical Pharmacology, Autonomic Dysfunction Service, Vanderbilt University, Nashville, TN USA; 5 Deutsches Zentrum für Herz-Kreislaufforschung (German Centre for Cardiovascular Research), Hamburg-Kiel-Lübeck, Germany; 6 Institute of Aerospace Medicine, German Aerospace Center (DLR) and Chair of Aerospace Medicine, Cologne, Germany

**Keywords:** Cardiovascular, Blood pressure, Pharmacological baroreflex, Chemoreflex, Phenylephrine

## Abstract

**Introduction:**

Baroreflexes and peripheral chemoreflexes control efferent autonomic activity making these reflexes treatment targets for arterial hypertension. The literature on their interaction is controversial, with suggestions that their individual and collective influence on blood pressure and heart rate regulation is variable. Therefore, we applied a study design that allows the elucidation of individual baroreflex–chemoreflex interactions.

**Methods:**

We studied nine healthy young men who breathed either normal air (normoxia) or an air–nitrogen–carbon dioxide mixture with decreased oxygen content (hypoxia) for 90 min, with randomization to condition, followed by a 30-min recovery period and then exposure to the other condition for 90 min. Multiple intravenous phenylephrine bolus doses were applied per condition to determine phenylephrine pressor sensitivity as an estimate of baroreflex blood pressure buffering and cardiovagal baroreflex sensitivity (BRS).

**Results:**

Hypoxia reduced arterial oxygen saturation from 98.1 ± 0.4 to 81.0 ± 0.4% (*p* < 0.001), raised heart rate from 62.9 ± 2.1 to 76.0 ± 3.6 bpm (*p* < 0.001), but did not change systolic blood pressure (*p* = 0.182). Of the nine subjects, six had significantly lower BRS in hypoxia (*p* < 0.05), two showed a significantly decreased pressor response, and three showed a significantly increased pressor response to phenylephrine in hypoxia, likely through reduced baroreflex buffering (*p* < 0.05). On average, hypoxia decreased BRS by 6.4 ± 0.9 ms/mmHg (19.9 ± 2.0 vs. 14.12 ± 1.6 ms/mmHg; *p* < 0.001) but did not change the phenylephrine pressor response (*p* = 0.878).

**Conclusion:**

We applied an approach to assess individual baroreflex–chemoreflex interactions in human subjects. A subgroup exhibited significant impairments in baroreflex blood pressure buffering and BRS with peripheral chemoreflex activation. The methodology may have utility in elucidating individual pathophysiology and in targeting treatments modulating baroreflex or chemoreflex function.

## Introduction

Arterial baroreflexes and peripheral chemoreflexes have been implicated in the pathogenesis of arterial hypertension and identified as treatment targets [1]. Both originate from receptors, namely, the carotid sinus baroreceptors and carotid body chemoreceptors, respectively, located in close proximity to the carotid bifurcation [2, 3]. Baroreflexes buffer changes in blood pressure through counter-regulatory adjustments in efferent sympathetic and parasympathetic activity and affect long-term blood pressure control [4], and carotid body chemoreceptor stimulation raises sympathetic activity [5]. In arterial hypertension, the sympathetic baroreflex gain is depressed and the baroreflex is reset to higher blood pressure [6]; in contrast, carotid chemoreceptors are sensitized [7–9] and generate tonic sympathetic activation [10–13] in hypertensive patients. Baroreceptor and carotid body afferents converge onto neurons in the dorsomedial medulla [14], resulting in an inhibitory interaction [15, 16]. Thus, impaired baroreflex regulation in arterial hypertension may be mediated in part through inappropriate carotid body chemoreceptor activation [1, 9]. Electrical carotid sinus stimulators and stents augmenting baroreceptor transmission as well as peripheral chemoreceptor modulation have been developed for hypertension management; however, all baroreceptor reflex modulation procedures developed to date exhibit an unacceptably high proportion of non-responders [17–20]. Blood pressure reductions following such interventions are likely determined by interindividual differences in baroreflex–chemoreflex interactions, which are not uncovered through routine clinical testing. While human baroreflex–chemoreflex interactions have been previously investigated [16, 21, 22], we hypothesized that a combination of physiological profiling and multiple repeated measurements could be utilized to elucidate individual baroreflex–chemoreflex interactions. In our study, hypoxia served as the peripheral chemoreceptor stimulus. In a similar fashion, patients have been repeatedly exposed to test and control interventions, such as statin and placebo treatment, to elucidate individual responses in so-called N-of-one trials [23, 24].

## Methods

### Subjects

Nine healthy young men with a mean age of 28.8 ± 3.0 years and a mean body mass index of 23.8 ± 0.8 kg/m^2^ were enrolled in the study (Table 1). Medical history and results from the physical examination, resting electrocardiogram, spirometry, and routine blood testing were all in the normal range. The ethics committee of the North Rhine medical association approved the study, and the study was registered in the German Clinical Trials register (DRKS00013101). Written informed consent was obtained from each participant before study entry.

**Table 1:** Baseline characteristics of subjects (*n* = 9)

Subject	SBP (mmHg)	DBP (mmHg)	HR (bpm)	BMI (kg/m^2^)	Age (years)
01	126	78	61	27.4	37
02	136	78	61	24.5	30
03	122	74	65	22.6	24
04	132	82	66	24.5	38
05	136	70	67	24.2	20
06	125	80	79	21.2	22
07	139	82	48	24.3	34
08	127	62	80	20.8	24
09	127	72	55	22.6	33

Values in table are the baseline characteristics of each subject taken before the beginning of the first session

*SBP* Systolic blood pressure,* DBP* diastolic blood pressure,* HR* heart rate,* BMI* body mass index

### Protocol

All measurements were obtained with the subjects in the supine position after they had voided their bladder. We assessed non-invasive beat-to-beat blood pressure (Finapres® NOVA; Finapres Medical Systems, Enschede, The Netherlands), upper arm blood pressure, arterial oxygen saturation, end-tidal carbon dioxide (ETCO_2_) concentration, breathing frequency, tidal volume, minute ventilation (Innocor; Innovision, Odense, Denmark), and an electrocardiogram recording (Vital Guard 450C; Ivy Biomedical Systems, Inc., Branford, CT, USA). An antecubital venous catheter was set in place for drug administration. Baseline data were recorded for 10 min prior to starting the randomized protocol (Table 1).

**Fig. 1:**
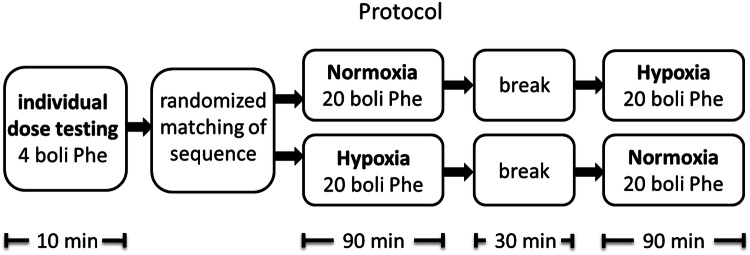
Schematic illustration of the study protocol.* Phe* Phenylephrine

We first determined the individual dose of the alpha-1-adrenoreceptor agonist phenylephrine that was needed to raise to blood pressure by 20–30 mmHg, using incremental doses of between 25 and 100 µg. All phenylephrine doses were immediately followed by a 10-ml saline flush. Both procedures were conducted in a standardized fashion using an automated bolus injector (Accutron MR; Medtron AG, Saarbrücken, Germany). Bolus flow rates were 5 ml/s for phenylephrine and 2.5 ml/s for the saline bolus, resulting in administration durations of 4.1–4.4 s. Following determination of the appropriate dose, we obtained repeated baroreflex measurements using the predetermined phenylephrine dose, with the subjects exposed to either the normoxic or hypoxic condition for 90 min, with randomization to one or the other condition and single blinding (subjects). Following the first exposure, subjects were allowed to recover for 30 min during which time they could stand up and empty their bladder, with the aim to minimize any carry-over effects from being supine or hypoxic, before being exposed for 90 min to the other condition. In the first 10 min of each exposure, inspiratory oxygen was slowly lowered to attain hypoxia or kept constant for normoxia testing. Thereafter, 20 phenylephrine bolus doses were applied, one every 4 min. The study protocol is illustrated in Fig. 1.

### Hypoxic peripheral chemoreflex stimulation

We utilized a hypoxic gas mixture containing nitrogen, oxygen, and carbon dioxide. Compressed air and nitrogen were mixed in a blender (Bird™ Blender low flow; Vyaire Medical Inc., Mettawa, IL, USA). Carbon dioxide was added separately in order to maintain normocapnia. Gas flows were controlled using flowmeters and buffered in a Douglas bag from which the subject could inhale without resistance or pressure via a face mask. For normocapnic hypoxia, the target was 80% arterial oxygen saturation, achieved by manual adjustment. To keep subjects blind to the condition, the same toolset was used to apply compressed air during both the hypoxia and normoxia procedures.

### Baroreflex measurements

Two parameters that characterize baroreflex function were assessed, namely, cardiovagal baroreflex sensitivity (BRS) and baroreflex buffering capacity. For both measurements we utilized the increase in finger blood pressure after the administration of each phenylephrine bolus. This pharmacological approach ensures greater independence from ventilatory influences on baroreflex function than methods based on spontaneous variations in hemodynamic variables. The investigator was blinded to the condition, i.e., normoxia or hypoxia.

1. Cardiovagal BRS was assessed as the slope of the regression line between each systolic blood pressure (SBP) value and the following RR interval during the phenylephrine-induced pressure rise without an offset [25, 26]. The slope was accepted if Pearson’s correlation coefficient *r* > 0.5 [27].
2. Larger increases in blood pressure after the administration of a defined phenylephrine bolus denote reduced the baroreflex buffering capacity. We determined this phenylephrine sensitivity as rise in SBP (ΔSBP) from the baseline before each bolus to the SBP maximum after bolus application [28]. Baseline SBP was calculated by averaging ten readings taken before each bolus. Maximum SBP was obtained by averaging the three highest consecutive SBP values within 60 s after each bolus.

### Statistics

Individual BRS and phenylephrine responses were analyzed using unpaired *t* tests. For group comparisons, we used medians of up to 20 boli per condition in order to minimize the effect of possible outliers and analyzed these via the paired *t* test. Significance was assumed at *p* < 0.05. If not otherwise indicated, all values were expressed as the mean ± SE. Power for BRS and change in SBP were calculated post hoc by means of the statistical analysis tool G*Power 3.1. We estimated the number of bolus repetitions needed to estimate individual effects between normoxia and hypoxia with receiver operating characteristic curve analysis performed with SPSS V.21 software (IBM Corp., Armonk, NY, USA). All other statistical analyses were done using Graphpad Prism 7.04 (GraphPad Software Inc., San Diego, CA, USA)

## Results

All subjects tolerated baroreflex testing in both normoxia and hypoxia, and no adverse events were observed. Arterial oxygen saturation was 98.1 ± 0.4% during normoxia and 81.0 ± 0.4% during hypoxia (*p* < 0.001), and ETCO_2_ remained at baseline values (38.31 ± 0.9 [normoxia] vs. 37.78 ± 1.1 mmHg [hypoxia]; *p* = 0.245). Respiratory rate (16 ± 1 vs. 16 ± 1/min; *p* = 0.588) did not change. During hypoxia, tidal volume increased from 0.77 ± 0.04 to 0.95 ± 0.07 l (*p* = 0.002), and minute ventilation increased from 11.86 ± 0.51 to 13.95 ± 0.49 l/min (*p* = 0.036); the mean respiratory chemoreflex response (ΔVe/SpO_2_) was 11.79 ± 4.80 (l/min)/%.

**Fig. 2:**
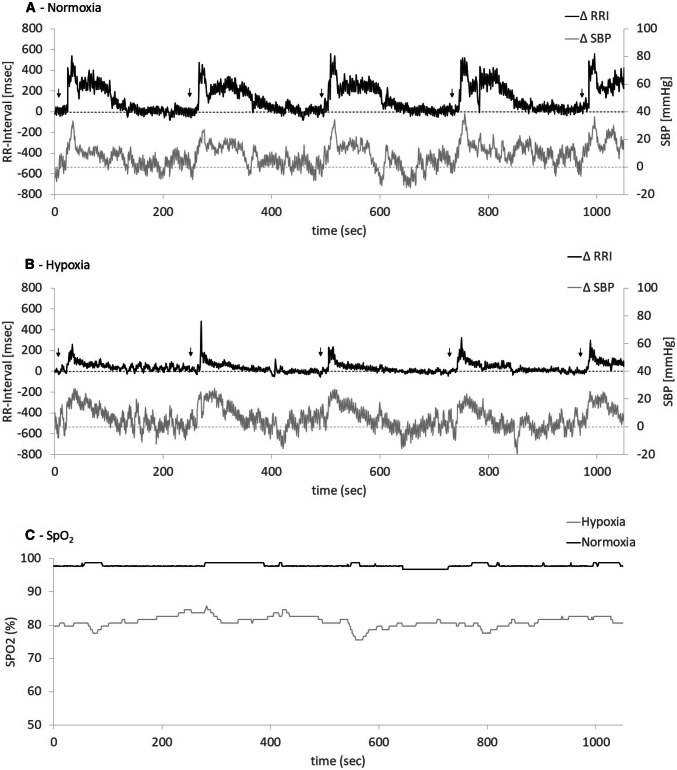
Original recording of RR-interval (*RRI*) and systolic blood pressure (*SBP*) responses to phenylephrine boli given during normoxia (**a**) and hypoxia (**b**), as well as corresponding peripheral capillary oxygen saturation (*SpO2*) curves (**c**).** a**,** b** Black arrows show timepoints at which phenylephrine boli were injected; the black upper lines correspond to RRI with the RRI axis on the left, and the lower gray lines correspond to SBP with the axis on the right side

Blood pressure was 133 ± 3/76 ± 2 mmHg during normoxia and 135 ± 3/76 ± 2 mmHg during hypoxia. During the experiment systolic blood pressure ranged from 122 to 154 mmHg during normoxia and from 121 to 148 mmHg during hypoxia. Heart rate increased from 63 ± 2 bpm during normoxia to 76 ± 4 bpm during hypoxia (*p* < 0.001). Figure 2 illustrates original blood pressure and RR-interval responses to repeated phenylephrine doses during normoxia and during hypoxia in one subject.

**Fig. 3:**
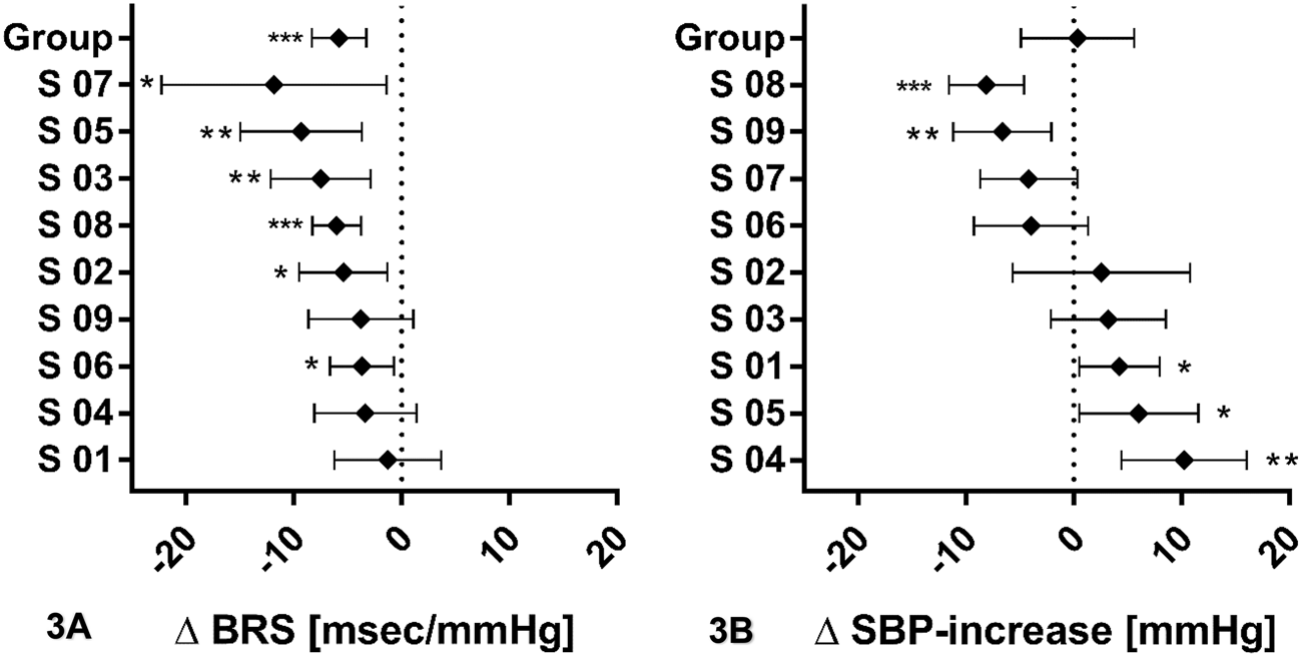
Forest plots of individual changes (hypoxia–normoxia) in cardiovagal baroreflex sensitivity (*BRS*) (**a**) and individual changes in phenylephrine sensitivity (**b**). Data points indicate mean difference in BRS or SBP increase between hypoxia and normoxia, and error bars indicate the range of these differences (95% confidence interval). *p* values are shown next to each data point, and asterisks, where present, indicate significance of difference (*p < 0.05; **p < 0.01; ***p < 0.001)

Individual baroreflex responses are given in Tables 2, 3, and Fig. 3. Not all phenylephrine applications yielded responses suitable for baroreflex analysis. The number of bolus doses analyzed for each subject and condition ranged between 8 and 20 (Tables 2, 3). In hypoxia, BRS was significantly lowered in six subjects; Figure 3a illustrates individual changes in BRS, including the 95% confidence intervals, with reductions in BRS during hypoxia in these six subjects. Overall BRS decreased from 19.9 ± 2.0 ms/mmHg in normoxia to 14.1 ± 1.6 ms/mmHg in hypoxia (*p* < 0.001; power 0.995; Fig. 3a).

**Table 2:** Individual baroreflex responses of the nine subjects in normoxia and hypoxia

Subject	Order of condition applied (condition applied first)	Normoxia		Hypoxia		Statistical analysis	
		BRS (ms/mmHg)	Boli (*n*)	BRS (ms/mmHg)	boli (*n*)	*p *value	*t*
01	Hypoxia	19.6 ± 2.1	17	18.3 ± 1.4	19	0.603	0.525
02	Normoxia	14.9 ± 1.4	16	9.5 ± 1.4	17	0.011	2.689
03	Hypoxia	18.9 ± 1.9	18	11.4 ± 1.2	17	0.002	3.307
04	Normoxia	14.4 ± 1.2	19	11.1 ± 2.1	17	0.159	1.439
05	Normoxia	29.0 ± 2.0	14	19.6 ± 1.9	16	0.002	3.381
06	Hypoxia	14.7 ± 1.2	16	10.1 ± 0.9	17	0.017	2.533
07	Normoxia	28.6 ± 3.3	16	16.8 ± 2.6	8	0.028	2.35
08	Hypoxia	14.4 ± 0.8	20	8.3 ± 0.8	20	< 0.001	5.399
09	Hypoxia	24.9 ± 1.6	19	21.1 ± 1.8	17	0.122	1.585

*BRS* Baroreflex sensitivity

BRS values are presented as the individual mean values ± standard error of the mean (SEM), together with the corresponding number of boli

*p* values and *t* correspond to measurements in normoxia vs. those in hypoxia. Significance was assumed at *p* < 0.05

**Table 3:** Change in systolic blood pressure in normoxia and hypoxia in the nine subjects

Subject	Order of condition applied (condition applied first)	Normoxia		Hypoxia		Statistical analysis	
		ΔSBP (mmHg)	Boli (*n*)	ΔSBP (mmHg)	Boli (*n*)	*p* value	*t*
01	Hypoxia	17.6 ± 1.2	20	21.8 ± 1.4	20	0.026	2.31
02	Normoxia	25.7 ± 2.7	20	23.2 ± 2.9	17	0.531	0.633
03	Hypoxia	27.8 ± 1.1	20	31.0 ± 2.4	20	0.232	1.214
04	Normoxia	19.4 ± 1.7	19	29.7 ± 2.3	18	0.001	3.576
05	Normoxia	18.1 ± 1.6	19	24.1 ± 2.2	20	0.033	2.21
06	Hypoxia	30.0 ± 1.7	20	26.0 ± 2.0	20	0.1396	1.509
07	Normoxia	17.1 ± 1.8	20	13.0 ± 1.4	20	0.069	1.872
08	Hypoxia	30.3 ± 1.3	20	22.2 ± 1.1	20	< 0.001	4.714
09	Hypoxia	19.1 ± 1.2	20	12.5 ± 1.9	20	0.006	2.943

ΔSBP values are presented as individual mean values ±  SEM, together with the corresponding number of boli

*p* values and *t* correspond to measurements in normoxia vs. those in hypoxia. Significance was assumed at *p* < 0.05

Five subjects showed a significant change in phenylephrine responsiveness with hypoxic peripheral chemoreflex stimulation (Table 3), with phenylephrine responsiveness significantly decreasing in two subjects and significantly increasing in three subjects. In the other four subjects, phenylephrine sensitivity did not change significantly. Overall, phenylephrine responsiveness did not change (*p* = 0.878), as shown in Fig. 3b, which plots individual changes in phenylephrine sensitivity, including the 95% confidence intervals, and indicates the high interindividual variability.

The optimal number of repetitions for individual BRS changes was ten boli with a sensitivity of 0.96. This is derived from our heathy subjects with an effect size of 6 ± 3 ms/mmHg (difference in BRS between normoxia and hypoxia).Overall, we did not observe a linear correlation between changes in BRS or phenylephrine responsiveness and respiratory parameters (respiratory rate, tidal volume, or minute ventilation). Individual respiratory parameters varied, as shown in Table 4.

**Table 4:** Respiration parameters recorded for each of the nine subjects throughout each session in normoxia or hypoxia

Subject	Respiratory rate (breaths/min)		Tidal volume (l)		Minute ventilation (l/min)		ΔSpO_2_ (%)	ΔVe/ ΔSpO_2__2_ ([l/min]/%)
	Normoxia	Hypoxia	Normoxia	Hypoxia	Normoxia	Hypoxia		
01	9.53 ± 0.10	13.04 ± 0.08	1.05 ± 0.02	1.33 ± 0.01	9.33 ± 0.12	16.86 ± 0.11	17.30	43.53
02	17.11 ± 0.05	17.95 ± 0.13	0.80 ± 0.00	0.88 ± 0.01	13.64 ± 0.06	15.67 ± 0.15	18.51	11.11
03	14.13 ± 0.11	12.55 ± 0.16	0.87 ± 0.01	1.13 ± 0.02	11.62 ± 0.09	12.48 ± 0.14	16.53	5.25
04	16.30 ± 0.09	16.02 ± 0.09	0.73 ± 0.01	0.83 ± 0.01	11.64 ± 0.05	12.77 ± 0.10	16.40	6.91
05	19.12 ± 0.08	20.26 ± 0.08	0.69 ± 0.01	0.73 ± 0.01	12.53 ± 0.07	14.13 ± 0.08	16.86	9.48
06	19.13 ± 0.08	17.36 ± 0.12	0.73 ± 0.01	0.76 ± 0.01	13.66 ± 0.05	12.50 ± 0.09	15.29	− 7.57
07	18.89 ± 0.09	14.09 ± 0.13	0.74 ± 0.01	1.04 ± 0.02	13.17 ± 0.08	13.52 ± 0.20	16.89	2.05
08	18.29 ± 0.07	18.3 ± 0.09	0.62 ± 0.00	0.77 ± 0.00	11.13 ± 0.03	13.64 ± 0.05	19.03	13.20
09	14.15 ± 0.08	13.19 ± 0.08	0.72 ± 0.00	1.07 ± 0.01	10.02 ± 0.06	13.94 ± 0.08	17.74	22.11

*Ve* Minute ventilation,* SpO*_*2*_ peripheral capillary oxygen saturation,* ΔVe*/* ΔSpO*_*2*_ mean respiratory chemoreflex response

Where appropriate, values are given as the mean ±  SEM

Hemodynamic reactions were stable over time during normoxia and hypoxia (Fig. 2). The sequence of the conditions normoxia and hypoxia did not affect BRS or phenylephrine responsiveness measurements.

**Fig. 4:**
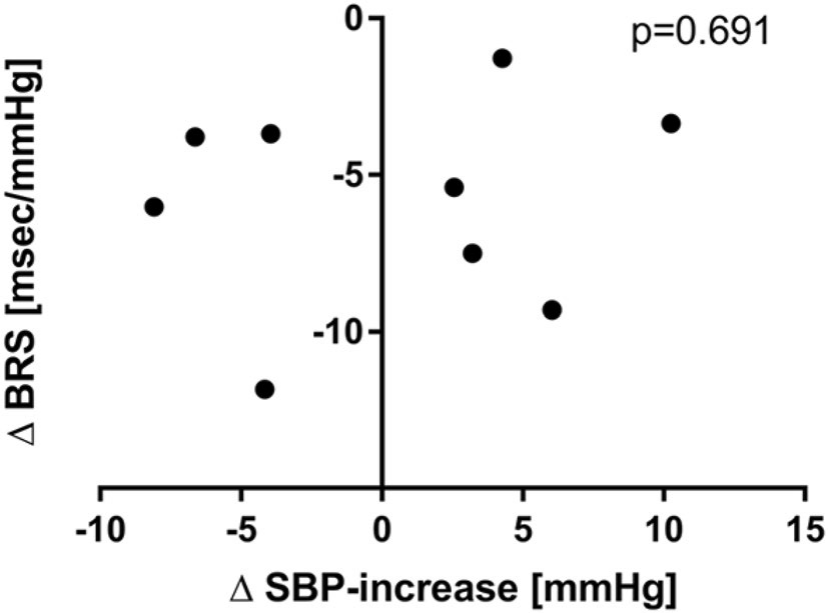
Correlation analysis between changes in cardiovagal baroreflex sensitivity and phenylephrine sensitivity. Phenylephrine sensitivity is considered as a marker of baroreflex-mediated buffering of blood pressure

We could not find a correlation between changes in cardiac BRS and baroreflex buffering capacity (Fig. 4).

## Discussion

In the study reported here, we applied a novel approach to elucidate individual baroreflex–chemoreflex interactions using repeated administrations of a standardized phenylephrine bolus during normoxia and hypoxia. While individual and group cardiovagal BRS responses to hypoxic peripheral chemoreceptor stimulation showed similar qualitative responses, individual responses exhibited substantial quantitative variability. Strikingly, individual phenylephrine responsiveness revealed qualitatively different responses, possibly resulting from changes in vascular sensitivity, in baroreflex blood pressure buffering, or in both mechanisms combined [28]. Our findings provide insight into human autonomic cardiovascular control mechanisms and could be useful in individualized assessment of baroreflex–chemoreflex interactions in patients with arterial hypertension, particularly in those considered for interventions targeting these reflexes.

The highly standardized assessment of baroreflex function using phenylephrine doses applied through an automated injector is a particular strength of our study. In previous studies, vasoactive drugs for baroreflex testing as well as subsequent saline flushes were manually applied [21, 29]. The lack of standardization likely introduces variability in drug delivery, immediate vascular responses, and subsequent baroreflex counter regulation. Our approach reduced this variability source. Another strength of our study is the large number of repeated phenylephrine tests which, unlike in previous studies, allowed for individual assessment of the baroreflex–chemoreflex interaction. We found that ten boli with a significant cardiovagal baroreflex response could be the appropriate number of boli for an optimal estimation of individual BRS changes induced by hypoxic peripheral chemoreceptor activation in the clinical setting.

We observed complex changes in baroreflex regulation with hypoxic peripheral chemoreflex stimulation. Average blood pressure was unchanged while the heart rate increased substantially. The baroreflex heart rate/pressure relationship was shifted towards higher heart rates and cardiovagal BRS was reduced. Increases in heart rate and maintained or increased blood pressure with peripheral chemoreflex stimulation have been previously described; in these studies sympathetic and parasympathetic baroreflex gains [21, 22, 30, 31] and baroreflex responsiveness [32] did not change. However, in two studies, chemoreflex-induced resetting of baroreflex-mediated sympathetic vasomotor control to higher blood pressure levels was observed using spontaneous baroreflex threshold [21] and modified Oxford techniques [29]. We speculate that the discrepancy in cardiovagal BRS responses may result in part from the methodology to assess baroreflex function or the high variability which we reduced through repeated testing. Interindividual and between-study variability in the BRS response to hypoxic chemoreceptor stimulation may partly result from variability in chemosensitivity [33].

Baroreflex regulation of sympathetic traffic to the vasculature is particularly relevant for blood pressure control. However, direct sympathetic nerve recordings are available in only a few medical centers and the tests often fail for technical reasons. As an alternative, the primary function of the baroreflex, which is to buffer blood pressure changes, can be assessed by applying vasoactive drugs before and during complete interruption of the baroreflex arc through ganglionic blockade [28]; the sensitivity to vasoactive drugs changes less in individuals with less efficient baroreflex blood pressure buffering than in those with more efficient baroreflex blood pressure buffering. Influences of gender, age, autonomic failure, and arterial hypertension on blood pressure buffering have been previously assessed [34–36]. Ganglionic blockers are no longer available for investigations on humans, and the methodology is cumbersome. Approximately 80% of the variability in systemic phenylephrine pressor sensitivity can be attributed to variability in baroreflex buffering rather than to vascular responsiveness [28]. That analysis included healthy persons and patients with various disorders known to affect vascular alpha-adrenoreceptor responsiveness. Therefore, phenylephrine responses without ganglionic blockade may provide an estimate of baroreflex buffering capacity. Thus, the results of our study may suggest that there is a dissociation between cardiovagal BRS and baroreflex blood pressure buffering during hypoxic chemoreflex stimulation.

The major limitation of our study is that findings in healthy subjects cannot be simply extrapolated to patients with arterial hypertension, particularly those considered for device-based treatments targeting the baroreflex or peripheral chemoreceptors. The fact that we did not assess sympathetic nerve activity is another potential limitation. Moreover, we determined an estimate of baroreflex blood pressure buffering rather than making a comparison phenylephrine responses before and after ganglionic blockade for the reasons mentioned above. While much of the interindividual variability in phenylephrine responsiveness can be explained by baroreflex blood pressure buffering, hypoxia-induced changes in vascular alpha-adrenoreceptor responsiveness may have confounded the analysis. However, data from human [37] and animal studies [38] regarding direct vascular actions of hypoxia are controversial, and it is evident that changes in vascular tone during hypoxia cannot be solely explained by baroreflex-mediated changes in adrenergic drive. Our observed BRS and phenylephrine responsiveness shift with hypoxia may be the result of hypoxia-induced vasodilation [39, 40]. Furthermore, hypoxia-induced heart rate changes are not abolished following bilateral carotid body removal [41]. However, in patients with resistant arterial hypertension, reductions in sympathetic traffic and blood pressure with electrical carotid sinus stimulation were found to be virtually identical with and without hypoxia [42]. This observation excludes major changes in the coupling between sympathetic activity and vasoconstriction with hypoxia. ETCO_2_, which affects cardiovascular control, was kept constant during our study. While we applied hypoxia in a steady-state fashion, transient hypoxia has been proposed to provide a more selective peripheral chemoreceptor stimulus [43]. Finally, hypoxia may have a prolonged effect on sympathetic activity [44]—although the sequence of normoxia and hypoxia sessions in our study did not affect our results. Moreover, we did not observe a substantial sustained effect of hypoxia on sympathetic activity or the response to electrical carotid sinus stimulation in patients with treatment-resistant arterial hypertension [42].

Despite these limitations, our study suggests that interindividual variability of baroreflex and chemoreflex regulation is so high that treatments modulating these mechanisms are exceedingly unlikely to be equally effective in all patients. In recent years there has been a thrust towards precision medicine [45]. Usually, the term implicates molecular assessment of a disease trait, such as somatic mutations in a specific cancer, that results in a targeted intervention. Outside oncology, the merits of the approach in common conditions, including arterial hypertension, have been limited; indeed, molecular signatures guiding hypertension treatment do not exist. However, individual physiological profiling could be utilized to identify underlying mechanisms driving hypertension and to direct treatments to patients more likely to respond. Our study suggests that repeated phenylephrine testing during normoxia and during hypoxia deserves to be tested in this setting.

## Data Availability

The raw data supporting the conclusions of this manuscript are available at the following link: https://figshare.com/s/5fbc8cff9925cec758d4. This includes the raw analog sampled data from beat-to-beat blood pressure, arterial oxygen saturation, ETCO_2_ concentration, breathing frequency, tidal volume, minute ventilation, and an electrocardiogram.
